# Oncostatin M expression and *TP53* mutation status regulate tumor-infiltration of immune cells and survival outcomes in cholangiocarcinoma

**DOI:** 10.18632/aging.103936

**Published:** 2020-11-07

**Authors:** Qi Liu, Tian Lan, Yuxuan Song, Jianpeng Cai, Xi Yu, Wei Chen

**Affiliations:** 1 Department of Pancreatico-Biliary Surgery, The First Affiliated Hospital, Sun Yat-Sen University, Guangzhou 510080, Guangdong Province, China; 2 Department of Urology, Tianjin Medical University General Hospital, Tianjin 300052, China

**Keywords:** cholangiocarcinoma, OSM, immune infiltration, TP53, prognosis

## Abstract

In this study, we used bioinformatics tools to analyze transcriptome data from cholangiocarcinoma (CCA) patients in multiple datasets (Sun Yat-sen University, TCGA and GSE32225 cohorts) to identify mechanisms that regulate tumor infiltration by immune cells and survival outcomes. We identified 96 differentially expressed genes (DEGs), including 13 upregulated and 83 downregulated genes, in CCA tissues as regulatory T cells were significantly higher and the proportions of activated natural killer cells and monocytes were significantly lower in CCA tissues than the precancerous tissues. The survival outcomes of CCA patients were associated with the *TP53* gene mutation status, levels of Oncostatin M (OSM) expression, and the proportions of tumor-infiltrating immune cell types, including dendritic cells, monocytes, and T follicular helper cells. Functional enrichment analysis of the DEGs in the high OSM-expressing CCA tissues showed that pathways related to tumor progression and immune response were significantly upregulated. Our study demonstrates that OSM expression and *TP53* mutation status regulate the tumor infiltration by immune cells and survival outcomes in CCA. OSM is thus a potential prognostic biomarker and therapeutic target in cholangiocarcinoma.

## INTRODUCTION

Cholangiocarcinoma (CCA) is a highly malignant tumor originating from the intra- or extra-hepatic bile ducts that has shown increasing morbidity and mortality rates worldwide in the last few years [1]. The primary treatment option for early-stage CCA patients is surgical resection, but the 5-year overall survival (OS) rate is below 10% because majority of the CCA patients are diagnosed in the advanced stages and are not amenable for surgery [2, 3]. Chemo-radio therapeutic outcomes are poor in CCA patients because the tumor is highly desmoplastic with fibrogenic connective tissue and immune cells such as T lymphocytes, natural killer (NK) cells and macrophages that infiltrate the tumor epithelium [4]. Hence, there is an urgent need to find effective targeted treatments to improve the clinical outcomes of CCA.

Advances in immunotherapy have shed greater focus on the role of tumor-infiltrating immune cells, which are vital components of the tumor immune microenvironment (TIME). CCA patients with a higher proportion of neutrophils and T-regulatory cells (Tregs) and lower proportion of CD8^+^ T cells in the tumor tissues are associated with poor prognosis [5]. However, only limited types of tumor-infiltrating immune cells have been analyzed in CCA tissues and the mechanisms regulating the tumor infiltration of immune cells are poorly understood.

Recent studies demonstrate that somatic mutations in the tumor tissues influence immunotherapeutic response in several cancers [6, 7]. Somatic mutations can reduce or abolish the ability of immune cells to recognize neoantigens on the tumor cells [8]. Some studies have reported that somatic mutations can influence immunotherapeutic outcomes [6, 7]. *TP53* is the most frequently mutated gene in more than 50% of all human cancers [9]. *TP53* mutations are also associated with the infiltration of immune cells into the tumor microenvironment [10–12]. In lung cancer patients, *TP53* gene mutation status is associated with prognosis and therapeutic outcomes [10]. TP53 expression is also a potential diagnostic biomarker in CCA patients [13].

Oncostatin M (OSM) is a cytokine secreted by differentiated histiocytic lymphoma cells [14]. OSM regulates the secretion of cytokines such as IL-6, G-CSF and GM-CSF from the endothelial cells [15–17]. OSM also inhibits growth and proliferation of several types of tumor cells such as A375 melanoma [18]. Higher levels of OSM in the early stages of early gastric cancer and breast cancer regulate tumor progression [19, 20]. Altered OSM expression is also associated with 22q11-q13 somatic mutations in CCA [21]. However, the mechanism by which OSM expression regulates the growth and progression of CCA has not been reported.

In this study, we used bioinformatics tools to analyze the transcriptome data from multiple CCA patient datasets (Sun Yat-sen University, TCGA and GSE32225 cohorts) and determine the relationship between OSM expression, tumor infiltration of immune cell types, *TP53* gene mutational status and survival outcomes in CCA.

## RESULTS

Identification of differentially expressed genes in three CCA patient datasets

**Figure 1:**
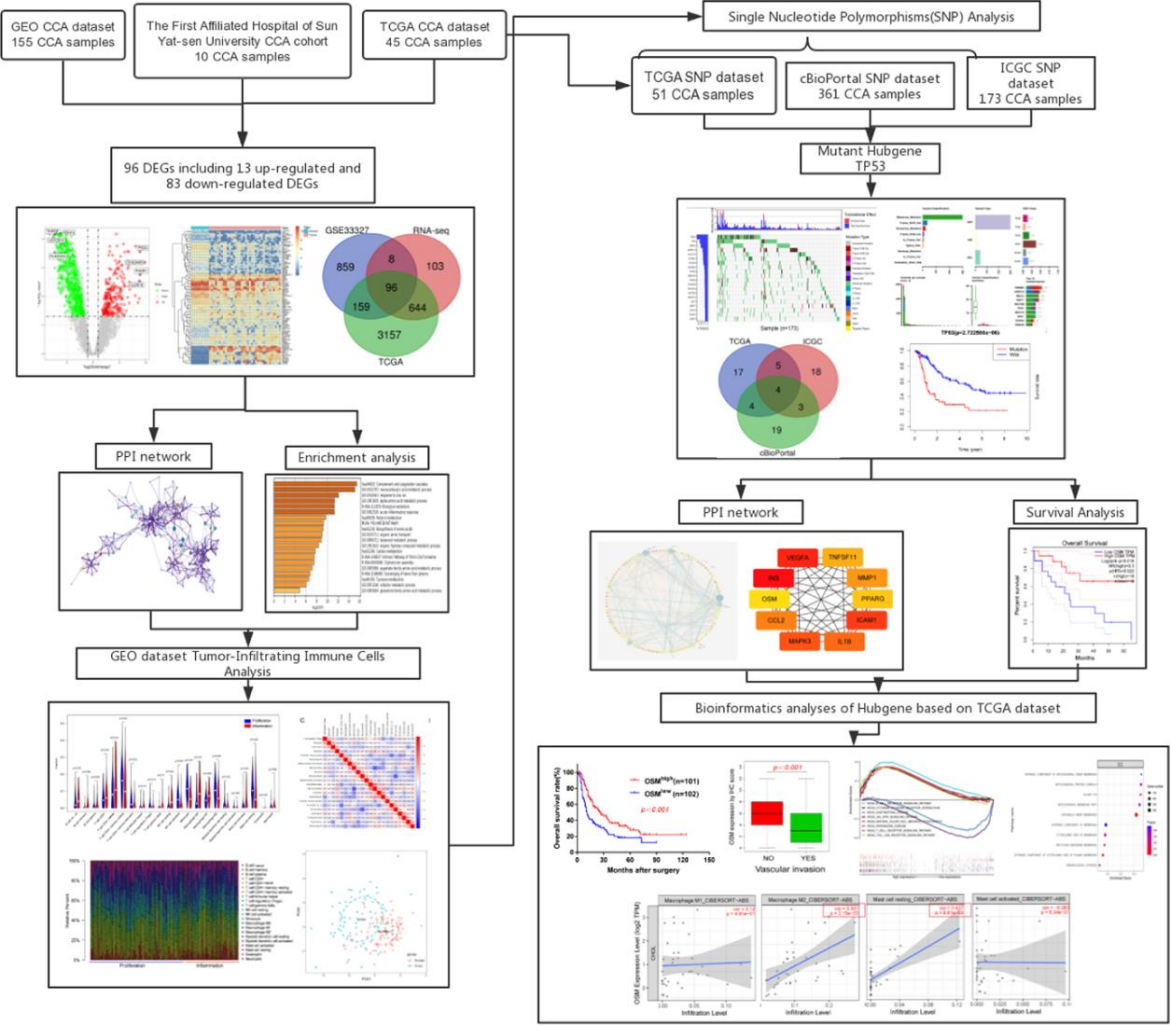
Flow chart of this research.

The study workflow diagram is shown in Figure 1.

### Identify the differentially expressed genes of three datasets

**Figure 2:**
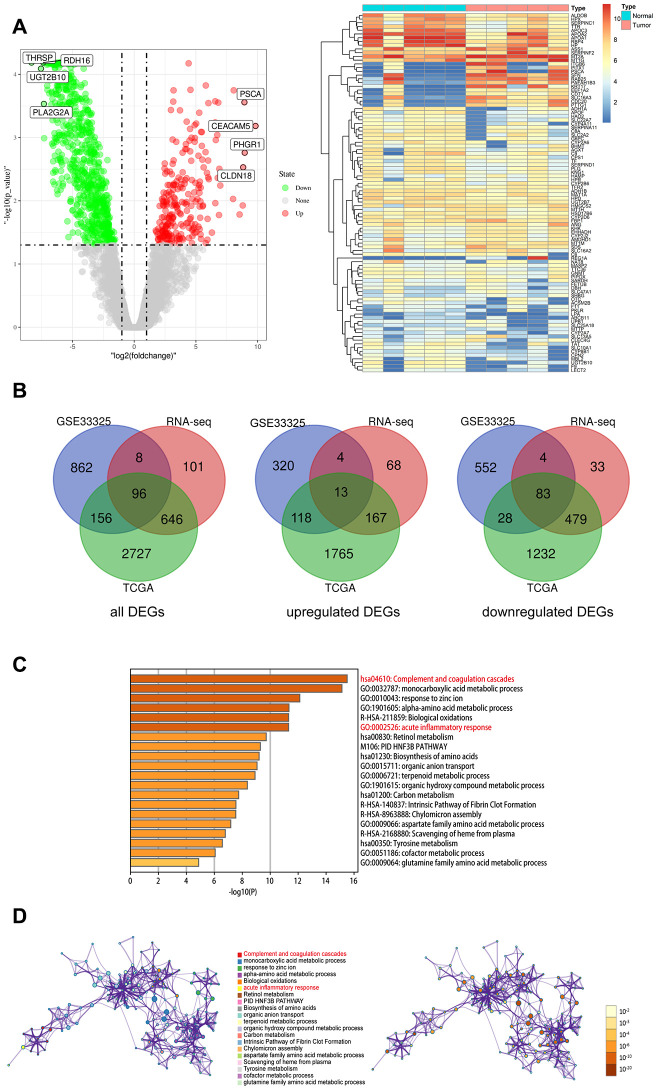
**Identify the differentially expressed genes (DEGs) of three datasets, and the pathway and process enrichment analysis of overlapped DEGs between CCA and precancerous tissues.** (**A**) Volcano plot and heatmap of 5 pairs matched CCA and precancerous tissues from our own RNA high-through sequencing data; (**B**) Venn diagram for overlapped DEGs of TCGA, GSE32225 and our own RNA high-through sequencing three datasets; (**C**) The top 20 reliable enrichment items of overlapped DEGs sort by -log10(P); (**D**) Network of the top 20 items and the p value of these items, each node with different color is an enriched item, the size of the node represents the degree of enrichment. And the p-value are ranked by the shades of color.

We first analyzed the RNA-seq data from various CCA patient datasets using the edgeR software to identify differentially expressed genes (DEGs) in CCA tissues. We identified 3625 DEGs (2063 upregulated and 1562 downregulated genes) in the TCGA CCA dataset, 1122 DEGs (455 upregulated and 667 downregulated genes) in the GSE32225 dataset, and 851 DEGs (252 upregulated and 599 downregulated genes) in the Sun Yat-Sen University CCA patient dataset (Figure 2A, Supplementary Figure 1). The volcano plots and heatmaps for all the DEGs are shown in Figure 2A.

### Inflammatory response and complement pathway genes are enriched in CCA tissues

We identified 96 overlapping DEGs between the three CCA patient datasets, including 13 upregulated and 83 downregulated genes using BEG online tools (Figure 2B). Functional and pathway enrichment analysis of the DEGS using Metascape showed that genes related to complement and coagulation pathway as well as acute inflammatory response were significantly enriched in the CCA patient tissues (Figure 2C, 2D).

### Landscape of tumor-infiltrating immune cells is altered in CCA tissues

**Figure 3:**
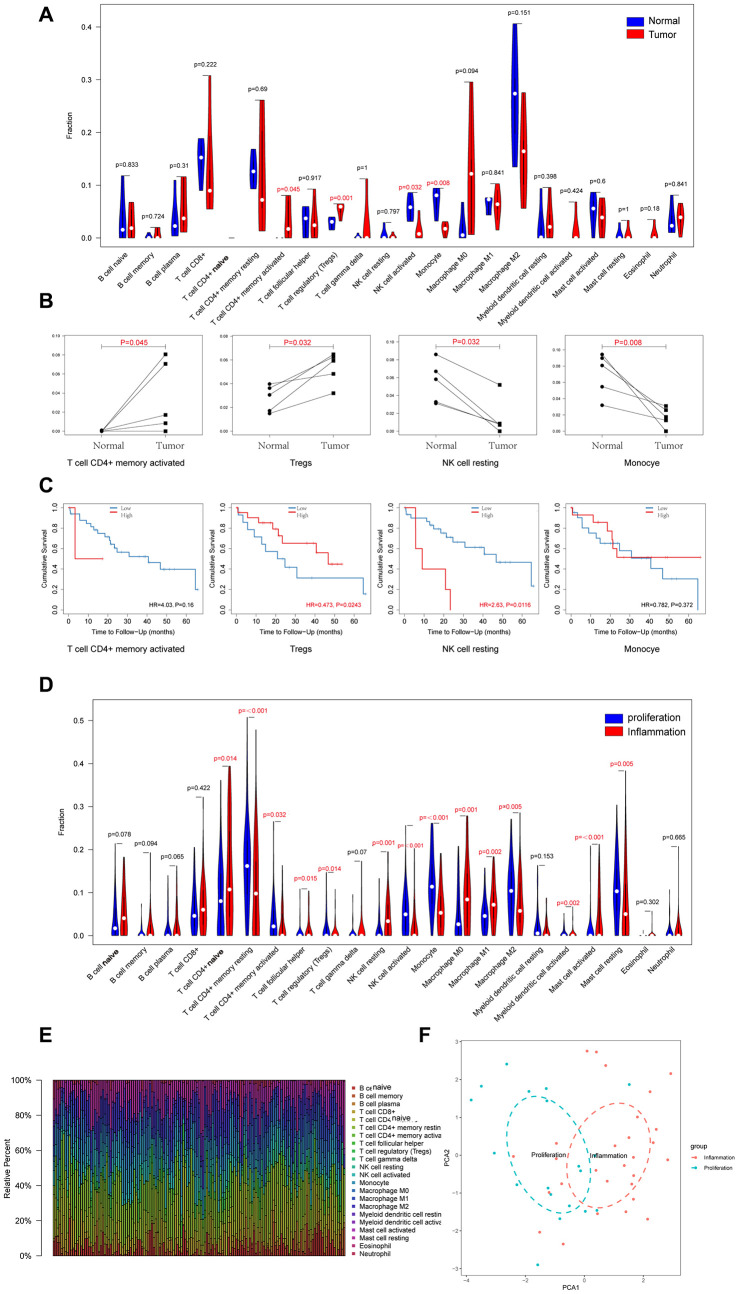
**Landscapes of Immune Cell Infiltration between CCA and Precancerous Tissues, and between Proliferation and Inflammation Types of CCA.** (**A**) 22 types of immune infiltrating cells in RNA high-through sequencing data between CCA and precancerous tissues; (**B**) Four types of immune infiltrating cells which are significant different between CCA and precancerous tissues; (**C**) Overall survival of four types of immune infiltrating cells which are significant different in CCA; (**D**) 22 types of immune infiltrating cells in GSE32225 data between proliferation and inflammation groups; (**E**) Stacked bar chart shows proportion of 22 immune infiltrating cells in each sample; (**F**) PCA (Principal components analysis) based on the content of 22 types of immune infiltrating cells indicated that inflammation and proliferation groups of CCA were generally distributed in two different directions in inflammation group and proliferation group respectively.

We used the CIBERSORT algorithm to analyze the proportions of 22 types of tumor-infiltrating immune cells in the CCA and precancerous tissues. As shown in Figure 3A, 3B, the proportions of CD4^+^ memory T cells (p=0.045) and T regulatory cells (Tregs; p=0.001) were significantly increased and the proportions of activated NK cells (p=0.032) and monocytes (p=0.008) were significantly reduced in the CCA tissues. Furthermore, high proportions of T regulatory cells had a better prognosis (HR=0.473, p=0.024), however, high proportions of NK cells had a poorer prognosis (HR=2.63, p=0.011).

Sia et al divided the CCA patient samples in the GSE32225 dataset into inflammation (n=57, 38%) and proliferation (n=92, 62%) groups [22]. Therefore, we analyzed the differences in the proportions of tumor-infiltrating immune cells between these two groups. The results showed that proportions of 14 out of 22 types of tumor-infiltrating immune cells analyzed were significantly different between the inflammation and proliferation groups of CCA patient tissues (Figure 3D, 3E). The proportions of CD4^+^ naïve T cells (p=0.014), follicular helper T cells (p=0.015), resting NK cells (p=0.001), M0 macrophages (p=0.001), M1 macrophages (p=0.002), activated myeloid dendritic cells (p=0.002), and activated mast cells (p<0.001) were significantly higher and the proportions of memory CD4^+^ resting cells (p<0.001), activated memory CD4^+^ cells (p=0.032) cells, T regulatory cells (p=0.014), activated NK cells (p<0.001), monocytes (p<0.001), M2 macrophages (p=0.005), and resting mast cells (p=0.005) were significantly lower in the inflammation group compared to the proliferation group of CCA patients. Furthermore, we generated visual plots of the relative proportions of 22 different types of immune cells in the tumor tissues from inflammation and proliferation groups of CCA patients using the ‘ggplot2’ R package and observed clear differences between the two groups (Figure 3F).

### *TP53* gene mutations are associated with survival outcomes in CCA patients

**Figure 4:**
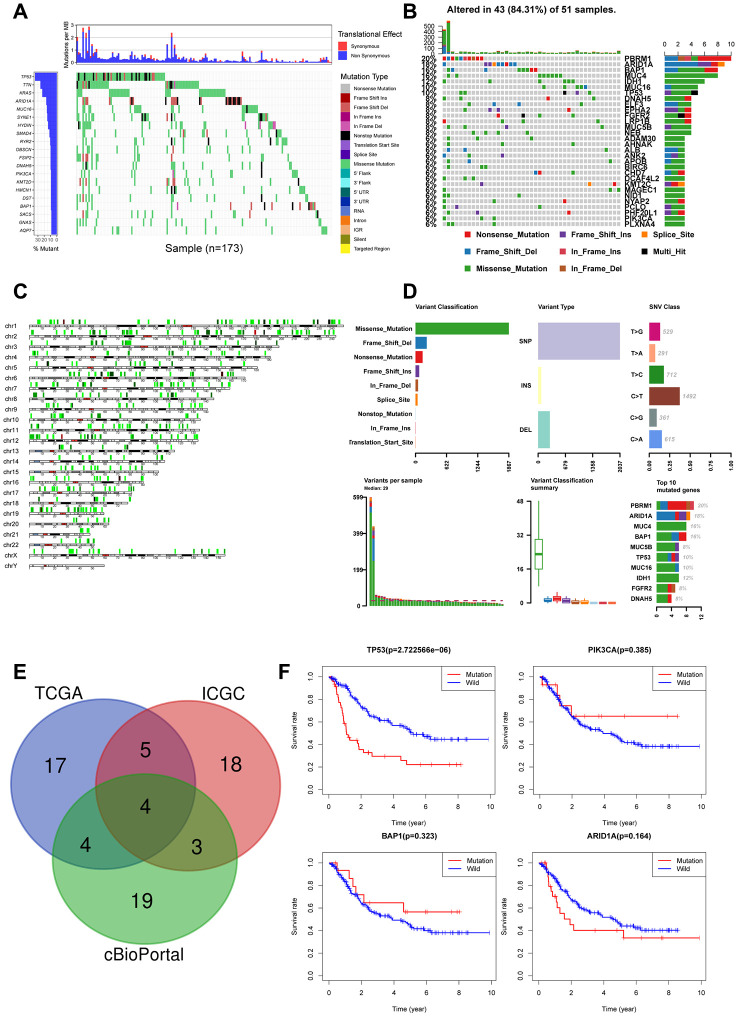
Landscape of somatic mutations in CCA (**A**) Waterfall plot depicts the frequencies and the mutation types of mutated genes in CCA from ICGC cohort. The left bar chart shows the TOP20 mutant genes frequencies of mutation. The bottom bar chart shows the mutation types; (**B**) Waterfall plot shows the mutated genes in CCA from TCGA cohort. The right bar chart shows the TOP30 mutant genes frequencies of mutation; (**C**) The positions of all the mutations in chromosomes, lower mutation frequency location is labeled with green color, while higher labeled with red; (**D**) Summary of mutation types of TCGA CCA dataset; (**E**) Venn diagram of overlapped top30 mutant genes of the three datasets respectively; (**F**) Overall survival analysis of four overlapped mutant genes.

We next explored the correlation between somatic mutations and tumor-infiltration of immune cells in the CCA tissues. We identified 20 frequently mutated genes in the ICGC cohort of 173 Japanese CCA patients (Figure 4A) and 30 frequently mutated genes in the TCGA cohort of American CCA patients (Figure 4B). The gene mutations were mostly missense type and were distributed on all the chromosomes (Figure 4C).

We analyzed the top30 mutant genes from all the three datasets and identified *TP53*, *PIK3CA*, *BAP1*, *ARID1A* as the most frequently mutated genes in the CCA tissues (Figure 4E). Kaplan-Meier survival curve analysis showed that overall survival was shorter for CCA patients with *TP53* mutations compared to those with wild-type *TP53* (Figure 4F). The other 3 genes, PIK3CA(p=0.385), BAP1(p=0.323), ARID1A(p=0.164) did not show prognostic significance. Hence, we focused on evaluating the correlation between *TP53* mutations and the tumor-infiltration of immune cells.

### Prognostic significance of OSM expression in CCA Patients with and without *TP53* gene mutations

**Figure 5:**
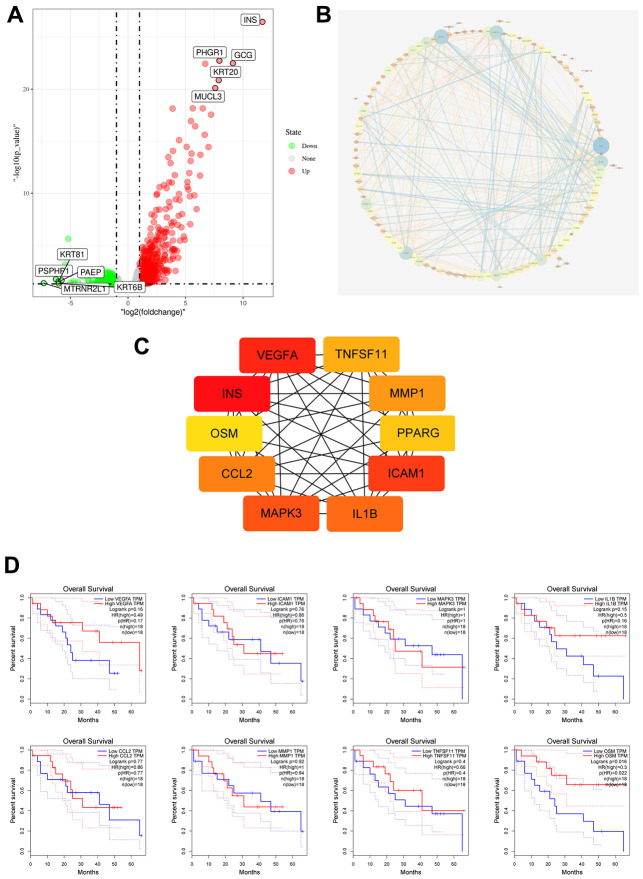
**Identification of Immune Related Hub Gene OSM of CCA Patients with and without TP53 Mutations.** (**A**) Volcano plot of TP53wild and TP53mutant group from TCGA dataset; (**B**) DEGs PPI network of TP53wild and TP53mutant group; (**C**) Top10 immune related genes were filtered from TP53 mutation associated DEGs by calculating the nodes scores of genes; (**D**) Overall survival of the Top10 immune related genes analyzed by KM curve analysis and log-rank test.

We analyzed 4 CCA patient samples with *TP53* mutations from 51 TCGA CCA patient samples with somatic mutations. and identified 879 DEGs, including 728 upregulated and 151 downregulated genes (p≤0.05) in the *TP53* mutant CCA patients (Figure 5A). PPI network analysis of DEGs using the STRING database showed that 133 DEGs were enriched for the GO term, immune system process (GO:0002376, p=0.007; Figure 5B). We then calculated the nodal scores using Cytoscape and identified 10 immune-related genes out of the 133 DEGs for further analysis (Figure 5C). Kaplan-Meier survival curve analysis and log-rank test showed that overall survival was significantly shorter for CCA patients with low OSM expression (OSM^low^ group) compared to those in the OSM^high^group (Figure 5D). The remaining 9 genes did not show prognostic significance. Hence, we selected OSM for further analysis.

### OSM expression is an independent predictor of prognosis in CCA patients

**Figure 6:**
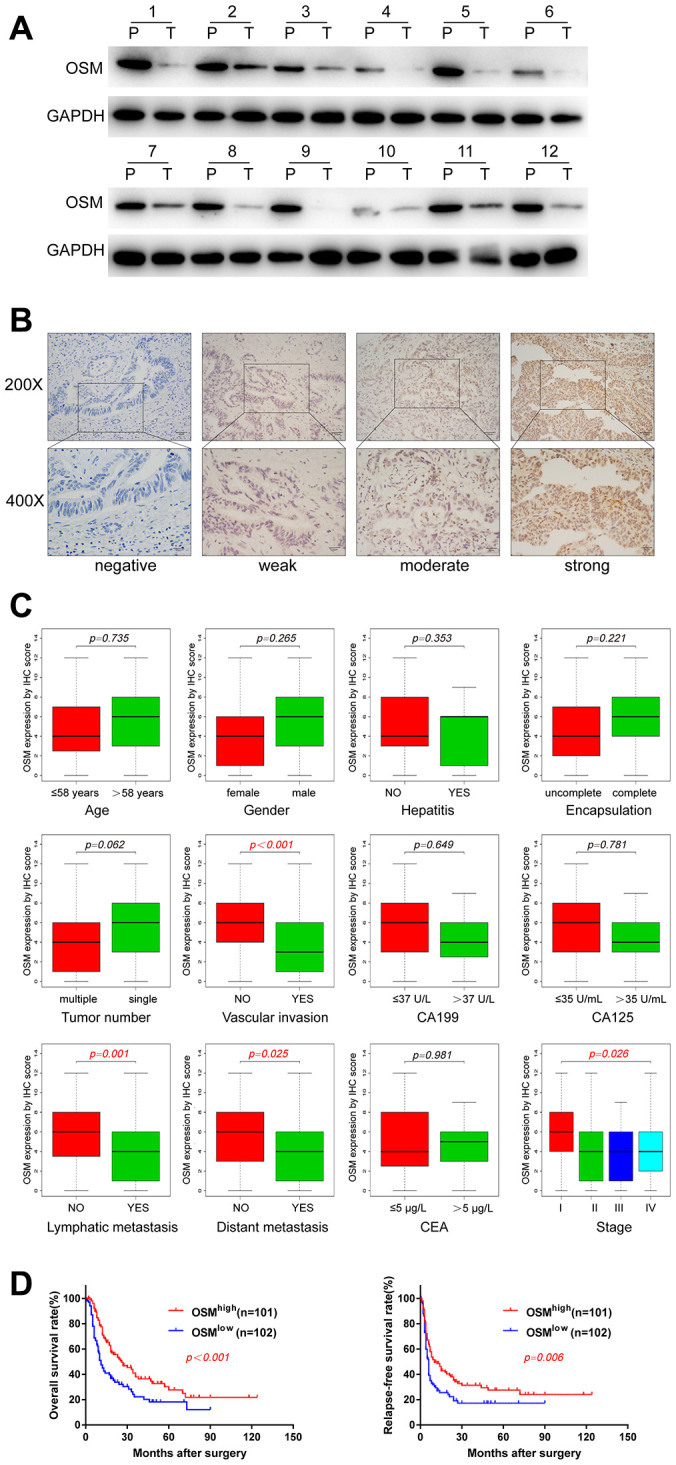
**OSM Expression Level in CCA And the Correlations with Clinical Characteristics.** (**A**) The protein expression of OSM in 12 paired CCA tumor (T) and adjacent non-tumor tissues (P) by western blotting. (**B**) Representative immunostaining images of OSM in CCA. (**C**) Boxplots depicted the correlation of OSM expression level and clinical characteristics; (**D**) Overall survival and relapse-free survival of OSM^high^ and OSM^low^ groups evaluated by IHC;

Western blot assays in 12 paired CCA tumor and adjacent non-tumor liver tissues showed OSM expression in CCA tumor tissues is lower than non-tumor liver tissues (Figure 6A). Then we analyzed the correlation between clinicopathological characteristics and the levels of OSM expression in CCA patients. Towards this, we analyzed the OSM expression in the Sun Yat-Sen university cohort of 203 CCA patients by IHC and divided the patients into high- and low-OSM expression groups (n=101 and n=102, respectively) using a median IHC expression score of 4 (Figure 6B). Kaplan-Meier survival curve analyses and log-rank test showed that the overall survival (OS) and disease-free survival (DFS) of the OSM^low^ group CCA patients was significantly shorter compared to the CCA patients from the OSM^high^ group (OS: p<0.001; DFS: p = 0.006; Figure 6D). These results demonstrate that high OSM expression indicates better prognosis in CCA. We then performed the Wilcox test, chi-square test and logistic regression analysis to evaluate the correlation between the levels of OSM expression and the clinicopathological characteristics of CCA patients. As shown in Figure 6C and Table 1, low expression of OSM correlates positively with lymphatic metastasis (chi-square test: p=0.003; Wilcox test: p<0.001), distant metastasis (chi-square test: p=0.045; Wilcox test: p=0.025), vascular invasion (chi-square test: p=0.001; Wilcox test: p<0.001), and tumor stage (chi-square test: p=0.033; Wilcox test: p=0.026).

**Table 1:** Correlation between OSM expression and clinical characteristics in 203 CCA patients by chi-square test.

**Variables**	**High (n=101)**	**Low (n=102)**	**P**
**Gender**			0.723
Male	54	52	
Female	47	50	
**Age**			0.529
>58	50	46	
≤58	51	56	
**Tumor**			0.726
>5cm	50	53	
≤5cm	51	49	
**Hepatitis**			0.834
YES	21	20	
NO	80	82	
**Tumor number**			<0.001
single	91	67	
multiple	10	35	
**Tumor capsulation**			0.136
none	40	51	
complete	61	51	
**CA199**			0.838
>37 U/L	55	57	
≤37 U/L	46	45	
**CA125**			0.919
>35 U/mL	32	33	
≤35 U/mL	69	69	
**CEA**			0.960
>5 μg/L	34	34	
≤5 μg/L	67	68	
**Tumor differentiation**			0.619
High	4	2	
Moderate	62	69	
Moderate-low	20	15	
Low	15	16	
**Lymphatic metastasis**			0.003
YES	29	50	
NO	72	52	
**Distant metastasis**			0.045
YES	16	28	
NO	85	74	
**Vascular invasion**			0.001
YES	10	27	
NO	91	75	
**AJCC 8^th^ TNM stage**			0.033
I	45	26	
II	12	17	
III	28	33	
IV	16	26	

Univariate Cox regression analysis showed that tumor numbers (HR=1.986, p<0.001), tumor encapsulation (HR = 0.510, p<0.001), CA199 levels (HR = 1.931, p<0.001), CA125 levels (HR = 1.995, p<0.001), CEA levels (HR = 1.741, p=0.002), tumor stage (HR =3.009, p<0.001), lymphatic metastasis (HR =2.507, p<0.001), distant metastasis (HR = 1.934, p=0.001), vascular invasion (HR = 2.409, p<0.001), and levels of OSM expression (HR = 0.533, p=0.001) were significantly associated with the OS of CCA patients. Moreover, tumor numbers, tumor encapsulation, CA199 levels, CA125 levels, CEA levels, tumor stage, lymphatic metastasis, distant metastasis, vascular invasion, OSM levels, and tumor size were associated with the relapse-free survival (RFS) of CCA patients (Table 2; all P≤0.05).

**Table 2:** Univariate and multivariate Cox analysis of potential prognostic factors in 203 CCA patients.

	**OS**			**RFS**	
	**HR (95%CI)**	***P* value**		**HR (95%CI)**	***P* value**
**Univariate COX analysis**					
Gender (male versus female)	1.162(0.823-1.642)	0.394		1.087(0.784-1.506)	0.616
Age (year) (>58 versus ≤58)	0.987(0.698-1.393)	0.939		0.940(0.678-1.303)	0.708
Tumor size (cm) (>5 versus ≤5)	1.321(0.935-1.867)	0.115		1.435(1.034-1.993)	**0.031**
Tumor number (multiple versus single)	1.986(1.377-2.862)	**0.000**		2.171(1.542-3.058)	**0.000**
Tumor encapsulation (complete versus none)	0.510(0.359-0.724)	**0.000**		0.652(0.468-0.906)	**0.011**
CA199(>37 U/L versus ≤ 37 U/L)	1.931(1.350-2.764)	**0.000**		1.468(1.053-2.046)	**0.023**
CA125 (>35 U/mL versus ≤35 U/mL)	1.995(1.402-2.839)	**0.000**		2.121(1.511-2.975)	**0.000**
CEA (>5μg/L versus ≤ 5μg/L)	1.741(1.219-2.487)	**0.002**		1.636(1.164-2.298)	**0.005**
Stage (IV+III+II versus I)	3.009(1.992-4.545)	**0.000**		2.875(1.955-4.227)	**0.000**
Lymphatic metastasis (yes versus no)	2.507(1.765-3.561)	**0.000**		2.119(1.517-2.961)	**0.000**
Distant metastasis (yes versus no)	1.934(1.298-2.880)	**0.001**		2.279(1.560-3.331)	**0.000**
Vascular invasion (yes versus no)	2.409(1.605-3.617)	**0.000**		1.762(1.180-2.631)	**0.006**
OSM (high vs low)	0.533(0.391-0.783)	**0.001**		0.644(0.463-0.896)	**0.009**
Liver cirrhosis (yes versus no)	0.628(0.386-1.021)	0.061		0.805(0.526-1.231)	0.316
**Multivariate COX analysis**					
Tumor number (multiple versus single)	1.611(1.079-2.407)	**0.020**		1.655(1.115-2.457)	**0.012**
Tumor encapsulation (complete versus none)	0.626(0.425-0.922)	**0.018**		0.731(0.509-1.049)	0.089
CA125 (>35 U/mL versus ≤35 U/mL)	1.322(0.888-1.968)	0.169		1.595(1.099-2.316)	**0.014**
CA199(>37 U/L versus ≤ 37 U/L)	1.519(1.037-2.226)	**0.032**		1.100(0.774-1.563)	0.594
CEA (>5μg/L versus ≤ 5μg/L)	1.300(0.868-1.948)	0.203		1.146(0.783-1.676)	0.483
Stage (IV+III+II versus I)	1.534(0.891-2.672)	0.121		1.770(1.063-2.947)	**0.028**
Lymphatic metastasis (yes versus no)	1.432(0.904-2.270)	0.126		1.194(0.771-1.850)	0.427
Distant metastasis (yes versus no)	0.921(0.582-1.457)	0.724		1.375(0.878-2.154)	0.164
Vascular invasion (yes versus no)	1.160(0.733-1.835)	0.527		0.978(0.618-1.549)	0.926
OSM (high versus low)	0.541(0.372-0.787)	**0.001**		0.745(0.518-1.073)	0.114

Multivariate Cox regression analysis showed that tumor numbers (HR=1.611, p=0.020), tumor encapsulation (HR = 0.626, p=0.018), CA199 levels (HR = 1.519, p=0.032) and the levels of OSM expression (HR = 0.541, p=0.001) were independent predictors of prognosis in CCA patients (Table 2). Furthermore, tumor numbers (HR=1.665, p=0.012), CA125 levels (HR = 1.595, p=0.014), and tumor stage (HR =1.770, P=0.028) were independent predictors of poor RFS (Table 2).

### OSM expression correlates with tumor infiltration of immune cells in CCA tissues

**Figure 7:**
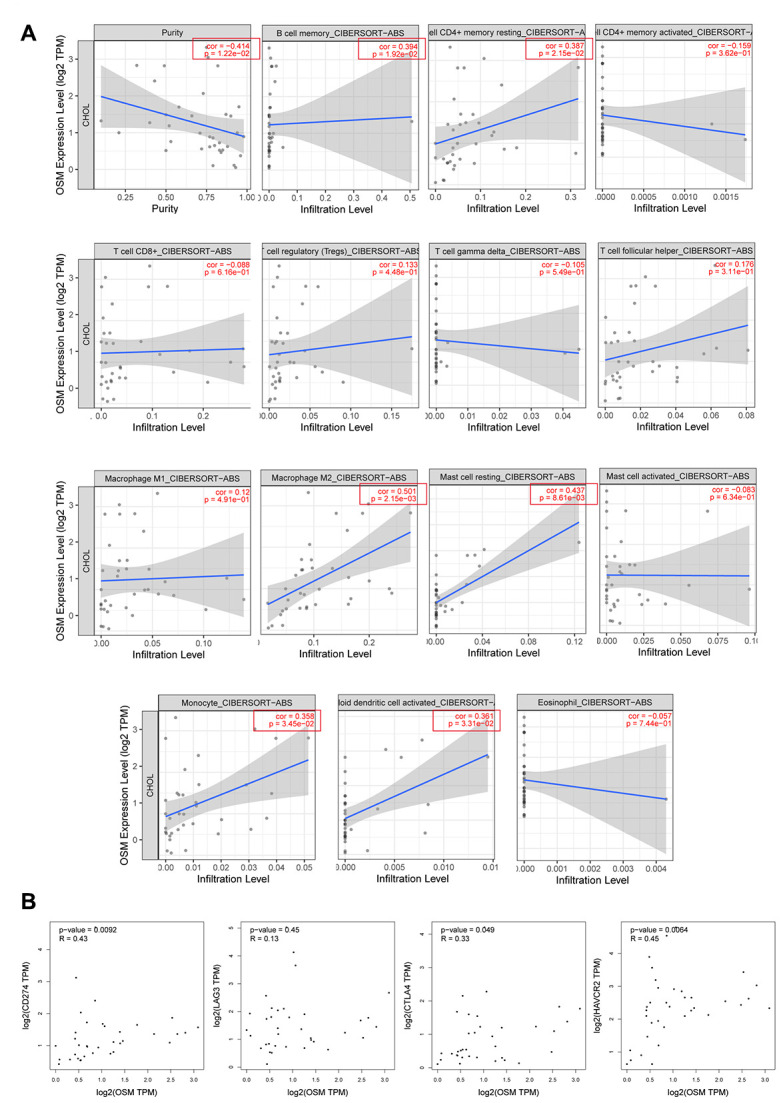
**The Correlations of OSM Expression Level and Immune Infiltrating Cells in CCA.** (**A**) The correlations with OSM expression level and the 15 types of immune infiltrating cells which are significant different between proliferation and inflammation groups of CCA; (**B**) The correlation between OSM expression and four immune checkpoints PD-L1 (CD274), CLTA4, LAG3, HAVCR2.

We then analyzed the correlation between the levels of OSM expression and the 14 types of tumor-infiltrating immune cells that are significantly different between the proliferation and inflammation groups of CCA patients. TIMER database analysis showed that OSM expression was positively associated with the proportions of memory B cells, resting CD4^+^ T cells, M2 macrophages, resting mast cells, monocytes, and activated myeloid dendritic cells (p<0.05; Figure 7A). We also analyzed the correlation between OSM expression and the proportion of cancer-associated fibroblasts and endothelial cells, which are known to regulate the tumor immune landscape. We observed no significant relationship between OSM expression and the proportion of cancer-associated fibroblasts (p=0.103) and endothelial cells (p=0.174) (Supplementary Figure 2).

We then analyzed the correlation between OSM expression and the immune cell markers in the CCA tissues relative to adjacent non-tumor liver tissues using the TIMER (Table 3) and GEPIA (Table 4) databases, respectively. The data were then adjusted for purity. The results showed a strong correlation between the levels of OSM expression and levels of markers related to DCs, monocytes, and Tfh cells in the CCA tissues (Tables 3, 4). Overall, these data suggest that OSM expression regulates the infiltration of immune cells such as DCs, monocytes, and Tfh cells into the tumor microenvironment in CCA tissues.

**Table 3:** Correlations between OSM and related genes and markers of immune cells in TIMER by spearman analysis.

**Cell type**	**Gene marker**	**None**		**Purity**	
		**Cor**	**P**	**Cor**	**P**
B cell	CD19	-0.084	0.631	-0.414	0.012
	CD22	0.233	0.177	-	-
	CD70	0.248	0.151	-	-
CD8+T Cell	CD8A	0.144	0.409	-	-
	CD8B	0.042	0.811	-	-
Tfh	IL21	0.008	0.964	-	-
	ICOS	0.355	**0.037**	-	-
	BCL-6	0.340	**0.046**	-	-
Thl	IL12RB2	0.228	0.188	-	-
	T-bet (TBX21)	0.085	0.628		
	IFN-γ (IFNG)	0.117	0.505		
	TNF-α (TNF)	0.219	0.207		
Th2	GATA3	0.159	0.363		
	STAT5A	0.120	0.493		
	IL13	-0.259	0.133		
Th17	IL17A	0.294	0.087	-	-
	STAT3	0.153	0.380	-	-
Treg	FOXP3	0.298	0.082		
	CCR8	0.204	0.240		
	STAT5B	0.148	0.396		
	TGFβ (TGFB1)	0.173	0.321	-	-
TAM	CCL2	0.289	0.092	-	-
	CD68	0.298	0.082	-	-
	IL10	0.291	0.089	-	-
M1 macrophages	NOS2	0.102	0.559	-	-
	ROS	0.121	0.488	-	-
M2 macrophages	ARG1	0.124	0.477	-	-
	MRC1	0.092	0.599	-	-
Monocyte	CD14	0.245	0.155	-	-
	CD33	0.384	**0.023**	-	-
NK	XCL1	0.066	0.708	-	-
	KIR3DL1	0.002	0.991	-	-
	CD7	0.135	0.440	-	-
Neutrophil	MPO	0.074	0.671	-	-
Dendritic cell	CD80	0.602	<**0.001**	-	-
	CD83	0.456	**0.006**	-	-
	CD86	0.500	**0.002**	-	-

**Table 4:** Correlations between OSM and genes markers of B cells, T helper cells, TAMs, monocytes, DCs and MDSCs in GEPIA by spearman analysis.

**Cell type**	**Gene marker**	**Tumor**		**Normal**	
		**Cor**	**P**	**Cor**	**P**
B cell	CD19	0.22	0.2	-0.46	0.21
	CD22	0.33	0.051	0.3	0.43
	CD70	0.4	**0.016**	-0.17	0.66
Tfh	CXCR5	0.046	0.79	-0.17	0.68
	ICOS	0.52	**0.001**	0.53	0.14
	BCL-6	0.31	0.064	0.67	0.059
Thl	IL12RB2	0.3	0.072	0.35	0.35
	T-bet (TBX21)	0.31	0.066		
	STAT4	0.2	0.24	0.05	0.91
Th2	CCR3	0.26	0.13	0.35	0.36
	CCR4	0.46	**0.005**	0.37	0.34
Th17	IL-23R	0.42	**0.012**	-0.028	0.94
	STAT3	0.19	0.25	0.57	0.12
Treg	FOXP3	0.41	**0.012**	-0.2	0.61
	IL-2Ra	0.45	**0.006**	0.084	0.83
TAM	CCL2	0.14	0.42	0.65	0.06
	CD68	0.21	0.22	0.23	0.54
	IL10	0.28	**0.095**	0.71	**0.034**
Monocyte	CD14	0.43	**0.009**	0.43	0.25
	CD33	0.46	**0.005**	-0.05	0.91
Dendritic cell	CD80	0.68	<**0.001**	0.47	0.2
	CD83	0.55	<**0.001**	0.37	0.34
	CD86	0.64	<**0.001**	0.63	0.076
MDSCs	CD33	0.46	0.005	-0.05	0.91
	CD11b	0.22	0.2	0.53	0.15

### OSM expression correlates with three immune regulatory checkpoints in CCA tissues

We then investigated the correlation between four immune regulatory checkpoints PDL1, CTLA4, HAVCR2, LAG3 and the levels of OSM expression in the CCA tissues by GEPIA. The correlation analysis method is spearman. Results showed that the expression of OSM in the CCA patient tissues showed strong correlation with CTLA4 (cor=0.33, p=0.049), HAVCR2 (cor=0.45, p=0.006), PDL1 (cor=0.43, p=0.009) in OSM-dependent manner (Table 5, Figure 7B), * means p≤0.05. And the correlations analysis results of OSM-related immune infiltrating cells monocytes, dendritic cells, Tfh cells with four immune regulatory checkpoints mentioned above were showed in Supplementary Table 1, * means p≤0.05. Monocytes and dendritic cells showed strong correlation with all these four checkpoints, but Tfh cells only showed correlation with CTLA4 and PDL1. This suggests that OSM expression modulates the efficacy of immunotherapy in CCA patients by influencing the immune regulatory checkpoints CTLA4, HAVCR2, PDL1, however, the mechanism is under further study.

**Table 5:** OSM expression correlates with four immune regulatory checkpoints in CCA tissue.

	**CTLA4**		**HAVCR2**		**LAG3**		**PDL1**	
	**Cor**	**P**	**Cor**	**P**	**Cor**	**P**	**Cor**	**P**
OSM	0.33	0.049	0.45	0.006	0.13	0.45	0.43	0.009

### Functional enrichment analysis of OSM-related genes in CCA tissues

**Figure 8:**
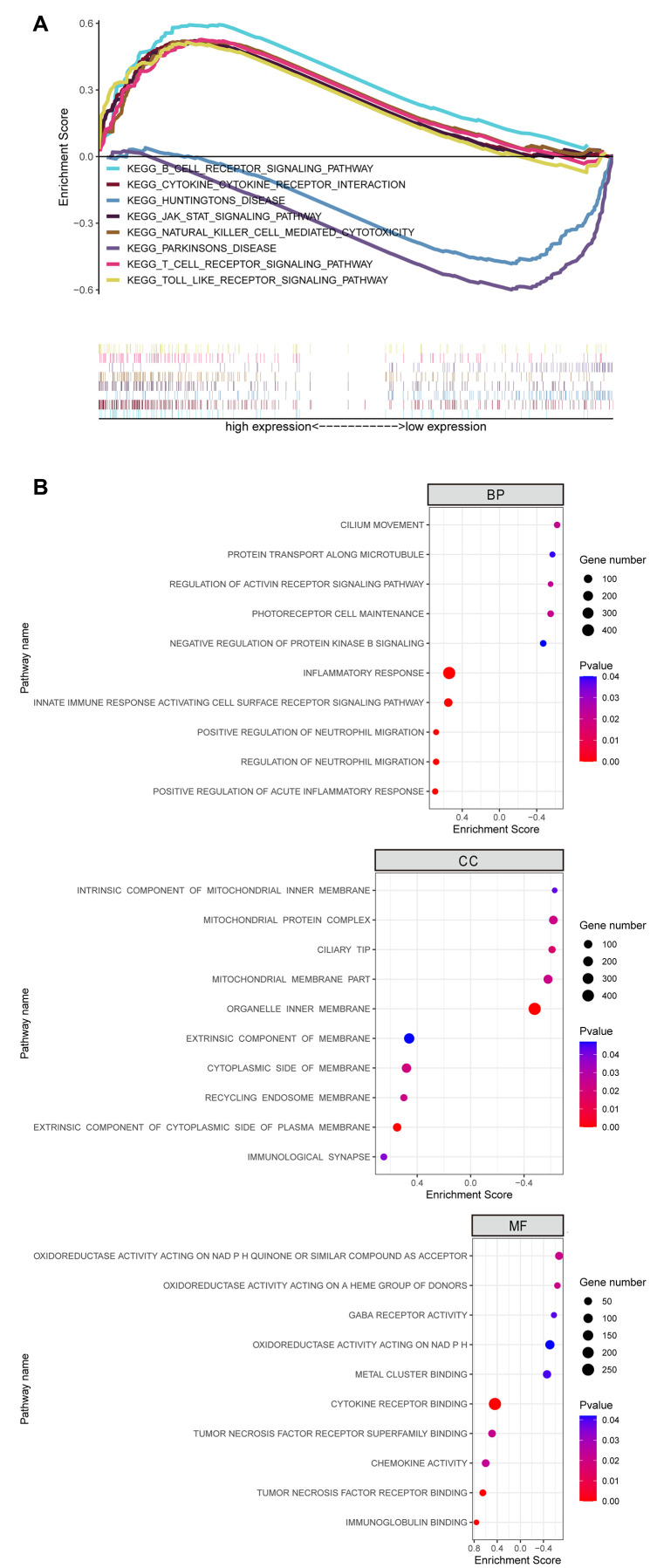
**GSEA Revealed Biological Function of Hub Gene OSM in CCA.** (**A**) KEGG pathway enrichment for OSM; (**B**) GO enrichment for OSM including biological process (BP), cell component (CC) and molecular function (MF).

We then performed functional enrichment analyses of OSM-related DEGs using data from OSM^high^ (n=18) and OSM^low^ (n=18) patients from the TCGA dataset. KEGG pathway analysis showed that pathways related to tumor progression and immune response, such as JAK-STAT signaling pathway, B-cell receptor (BCR), T-cell receptor (TCR), and TOLL-like receptor (TLR) signaling pathways, cytokine receptor signaling pathways, and natural killer (NK) cell-mediated cytotoxicity were significantly enriched in the OSM^high^ group, whereas, pathways related to Huntington’s and Parkinson’s disease were significantly enriched in the OSM^low^ group (Figure 8A). Furthermore, GO terms such as inflammatory response, regulation of neutrophil migration, innate immune response activating cell surface receptor signaling pathway, cell membrane and immunological synapse, GABA receptor activity, immunoglobulin binding, chemokine activity and cytokine receptor binding were significantly enriched in the OSM^high^ group (Figure 8B; Table 6).

**Table 6:** Gene Set Enrichment Analysis (GSEA) reveals biological function of OSM in CCA.

**GO/KEGG pathway Description**	**Gene Count**	**Enrichment Score**	***P Value***
**GO terms for biological process (BP)**			
Positive Regulation of Acute Inflammatory Response	28	0.69	<0.001
Innate Immune Response Activating Cell Surface Receptor Signaling Pathway	106	0.55	<0.001
Regulation of Neutrophil Migration	30	0.68	<0.001
Positive Regulation of Neutrophil Migration	26	0.68	<0.001
Inflammatory Response	440	0.54	<0.001
Cilium Movement	31	-0.62	0.021
Photoreceptor Cell Maintenance	34	-0.55	0.021
Regulation of Activin Receptor Signaling Pathway	25	-0.55	0.022
Protein Transport Along Microtubule	26	-0.57	0.039
Negative Regulation of Protein Kinase B Signaling	33	-0.47	0.04
**GO terms for cellular component (CC)**			
Recycling Endosome Membrane	40	0.5	0.023
Extrinsic Component of Cytoplasmic Side of Plasma Membrane	97	0.55	<0.001
Cytoplasmic Side of Membrane	167	0.48	0.021
Immunological Synapse	31	0.65	0.038
Extrinsic Component of Membrane	245	0.46	0.047
Ciliary Tip	42	-0.61	0.017
Mitochondrial Membrane Part	140	-0.58	0.023
Mitochondrial Protein Complex	108	-0.62	0.023
Organelle Inner Membrane	472	-0.48	<0.001
Intrinsic Component of Mitochondrial Inner Membrane	16	-0.63	0.043
**GO terms for molecular function (MF)**			
Immunoglobulin Binding	21	0.76	<0.001
Tumor Necrosis Factor Receptor Binding	29	0.65	<0.001
Chemokine Activity	46	0.6	0.023
Cytokine Receptor Binding	264	0.44	<0.001
Cytokine Receptor Activity	85	0.56	0.059
Oxidoreductase Activity Acting on NAD P H Quinone Or Similar Compound as Acceptor	52	-0.67	0.021
Oxidoreductase Activity Acting on A Heme Group of Donors	25	-0.64	0.021
Oxidoreductase Activity Acting on NAD P H	92	-0.51	0.042
Gaba Receptor Activity	22	-0.58	0.039
Metal Cluster Binding	61	-0.46	0.04
**KEGG signaling pathways**			
JAK Stat Signaling Pathway	151	0.52	<0.001
Toll Like Receptor Signaling Pathway	101	0.51	<0.001
Cytokine Receptor Interaction	257	0.52	0.019
Natural Killer Cell Mediated Cytotoxicity	131	0.52	0.018
T Cell Receptor Signaling Pathway	106	0.53	<0.001
B Cell Receptor Signaling Pathway	74	0.59	<0.001
Huntingtons Disease	161	-0.49	0.042
Parkinsons Disease	100	-0.61	0.08

## DISCUSSION

Improved understanding of the immunobiology of cancer tissues and the tumor microenvironment (TIME) has led to the emergence of highly effective anti-cancer therapies related to immune checkpoint blockers (ICB) such as PD1, PDL1, and CTLA-4 [23]. Currently, several studies are focused on identifying mechanisms that regulate tumor infiltration of immune cells because they modulate tumor progression an determine therapy and survival outcomes. The CIBERSORT algorithm is a useful tool to evaluate the proportions of 22 different types of tumor-infiltrating immune cells in all human cancers [24]. The tumor-infiltrating immune cells show a strong association with the prognosis of lung, bladder, and pancreatic cancer patients [10, 25, 26]. Several types of immune cells, such as lymphocytes, macrophages, neutrophils and natural killer cells, have been reported to regulate cholangiocarcinomagenesis [27]. Elevated levels of pre-operative peripheral blood neutrophil count relative to lymphocyte counts are associated with poor prognosis of intra-hepatic and extra-hepatic CAA patients [28]. CCA is a highly desmoplastic tumor with abundant amounts of various immune cell types, including tumor-associated macrophages (TAMs) and myeloid-derived suppressor cells or MDSCs [3]. TAMs interact with the cancer stem cell niche and modulate adhesion and invasive properties of tumor cells that are associated with tumor progression [29]. Our study demonstrates that the TAM numbers are significantly higher in the CCA tissues compared to the precancerous tissues.

The efficacy of ICB therapies is associated with the proportions of tumor-infiltrating immune cells. CCA patients with higher proportions of tumor-resident CD8^+^ T cells respond better to ICB therapies [3]. The expression of PD-L1 on the surface of TAMs significantly correlates with the density of CD3^+^- and CD8^+^-tumor-infiltrating lymphocytes (TILs) and the expression of human leukocyte antigen (HLA) class I molecules [10]. Furthermore, upregulation of PD-1 and PD-L1 in the tumor cells is associated with increased tumor invasiveness, poor prognosis, worse disease and metastasis-free survival, and lower infiltration of CD3^+^- and CD8^+^-TILs [11–13, 18]. In contrast, low expression of PD-L1 correlates with favorable prognosis in CCA patients [19]. This suggests that lymphocytic apoptosis induced by the PD-L1/PD-1 pathway promotes CCA progression.

The chemokines and other immune cell activation factors play an important role in the recruitment of tumor-infiltrating immune cells [23]. Our study demonstrates that the overall survival of extrahepatic cholangiocarcinoma (eCCA) patients significantly correlates with the proportions of DCs, neutrophils, and CD8^+^ T cells in the tumor tissues. Hence, the proportions of these three types of immune cells in the CCA tissues can potentially predict the prognosis of CCA patients. Moreover, enhanced infiltration of CD4^+^ and CD8^+^ T cells is associated with better overall survival, decreased lymph node metastases, and reduced venous and perineural invasion in CCA patients [5, 30, 31]. The neutrophils, CD8^+^ T cells, Tregs, and M2 macrophages also regulate inflammation during tumorigenesis [5]. However, the mechanisms regulating the differential infiltration of immune cell types in CCA are not clear. A recent study suggests that CCA cells increase the levels of TGF-β in the tumor tissues by activating Tregs, thereby inhibiting the immune response against CCA tumor cells [32].

Our study demonstrates significant differences in the proportions of activated CD4^+^ memory T cells, Tregs, resting NK cells and monocytes between the CCA and precancerous liver tissues. Studies have reported that CD4^+^ T cells inhibit tumor growth by secreting cytokines [33], and tumor-infiltrating CD4^+^ T cells are associated with increased overall survival of CCA patients [34]. Increased tumor-infiltration of Tregs is associated with worse DFS rates in the CCA patients [35]. Moreover, DFS positively correlates with the proportions of tumor-infiltrating neutrophils and tumor-associated macrophages in the CCA patients [5]. Preclinical data suggests that NK cells inhibit tumor growth by inducing apoptosis of CCA cells [36, 37]. Infiltration of TIE2-expressing monocytes (TEMs) combined with high Ang1 expression positively correlates with overall survival of hilar cholangiocarcinoma patients [38]. Furthermore, the proportions of different types of tumor-infiltrating immune cells vary significantly in the proliferation and the inflammation groups of CCA patients from the GSE32225 dataset [22].

Somatic gene mutations in the tumor cells affect the efficacy of immunotherapy [39]. Somatic mutations in the *EP300* gene activate the immune-regulatory signaling pathways and improve antitumor immunotherapeutic outcomes in bladder cancer patients [39]. We studied the correlation between somatic mutations in CAA tissues and tumor-infiltrating immune cells using the somatic mutation data for CCA tissues from the TCGA, cBioportal and ICGC databases, and observed that *TP53* gene mutations correlate with overall survival of CCA patients (Figure 4). Functional enrichment analyses showed that *TP53* mutant CCA patients were significantly enriched in pathways regulating immune cell functions. In lung cancer, genes related to *TP53* gene mutations are associated with tumor infiltration of immune cells [10]. We demonstrate that *TP53* mutation status correlates with the levels of OSM expression and the proportions of tumor-infiltrating immune cells in CCA tissues (Figure 7).

OSM is a cell growth regulating polypeptide that was first isolated from the serum-free supernatants of U937 histiocytic lymphoma cells [18]. OSM inhibits the proliferation and differentiation of liver cancer stem cells (LCSCs) and increases the sensitivity of liver cancer cells to the chemotherapeutic agent, 5-fluorouracil [40]. The role of OSM in cancers is controversial. OSM promotes epithelial to mesenchymal transition (EMT) of cancer stem cell (CSC) properties by activating Stat3/TGF-β/SMAD3 signaling, and therefore, promotes tumor metastasis and tumor recurrence [41]. OSM maintains hematopoietic progenitor cells in the bone marrow by regulating G-CSF and SDF-1 cytokine levels [14]. Bone marrow hematopoeisis is significantly reduced in the OSM-deficient mice because of elevated circulating levels of G-CSF [14]. Neutralizing anti-GM-CSF antibody reduces the levels of OSM secretion by the neutrophils, but anti-G-CSF antibody has no effects; moreover, co-culturing neutrophils with GM-CSF increases OSM secretion, but, co-culturing with G-CSF has no effects on OSM secretion [42]. OSM inhibits the proliferation of breast cancer cells and promotes their detachment and motility [42, 43]. IL-6 and IL-6 related cytokines promote OSM-dependent suppression of epithelial-specific genes and enhance epithelial cell death by phosphorylating STAT5 and STAT3 [42, 43]. OSM reduces the growth of human melanoma xenograft tumors in a mouse model in an IL-6-dependent manner [44]. In contrast, OSM and IL-6 are expressed in breast, prostate, and lung cancer cell lines, and promote tumor development by modulating lipid metabolism, matrix degradation, angiogenesis, and cellular dedifferentiation [45, 46]. In early stages of gastric cancer, higher OSM expression correlates with poor prognosis [19]. Furthermore, OSM plays an essential role in the early stages of breast cancer metastasis [20].

Our study has several limitations. Firstly, we did not have clinical data for the CCA patients from the GEO database. Furthermore, complete clinical data was available only for 30 CCA patients in the TCGA dataset, whereas, for other samples, only survival time and status were available. Therefore, we analyzed the correlation of OSM expression and prognosis of CCA with only limited number of clinicopathological characteristics. Secondly, we performed immunohistochemical analysis of the CCA patient samples from the Sun Yat-Sen University cohort to validate OSM expression and then investigated the correlation between clinicopathological characteristics and OSM expression based on those findings. However, further experiments such as RT-qPCR and functional assays are necessary to verify the conclusions of our study.

In conclusion, we demonstrate that tumor infiltration of immune cells is associated with OSM expression and the status of *TP53* gene mutations in CCA patients. We also demonstrate that OSM expression is an independent predictor of prognosis in CCA patients. Our study suggests that OSM expression is a novel prognostic biomarker and therapeutic target for CCA.

## MATERIALS AND METHODS

### CCA patient datasets

We enrolled 208 consecutive CCA patients that underwent hepatectomy between January 2007 and June 2016 at the First Affiliated Hospital of Sun Yat-sen University, Guangzhou, China. The inclusion criteria were: (1) patients diagnosed with CCA based on histology; (2) patients that underwent curative R0 resection of the primary tumor; and (3) clinicopathological information and follow-up data were available [47, 48]. Patients that received neoadjuvant chemotherapy or radiotherapy before hepatectomy were excluded. Therefore, we enrolled 203 out of 208 CCA patients for further analysis of clinicopathological characteristics and OSM immunohistochemistry. We also recruited 5 out of 208 patients between January 2019 and October 2019 for RNA high-throughput sequencing and 12 for western blotting. This study was approved by the Ethics Committee of the First Affiliated Hospital of Sun Yat-sen University. All recruited participants volunteered to participate in this study and signed an informed consent form before enrollment [49]. The clinicopathological characteristics of 17 participants are shown in the Supplementary Table 2.

We obtained transcriptome data from the TCGA database that included 9 precancerous and 36 CCA tissue samples and the Gene Expression Omnibus (GEO; http://www.ncbi.nlm.nih.gov/geo/) database (GSE32225 dataset with 6 precancerous and 149 CCA tissue samples). The CCA samples from the GSE32225 dataset were classified into inflammation and proliferation groups and used for analyzing immune cell infiltration. We also downloaded somatic mutation data for 51 American CCA patient samples from the TCGA portal, 173 Japanese CCA patient samples from the International Cancer Genome Consortium (ICGC) database (https://dcc.icgc.org/), and 361 CCA samples from the cBioportal database (https://www.cbioportal.org/). We obtained available clinical information of the CCA patients from the TCGA and ICGC databases for further analysis.

### RNA high-throughput sequencing

The RNA high-throughput sequencing of five matched primary tumor and precancerous tissues of CCA patients was performed using the Illumina HumanRef-8 WG-DASL v3.0 expression beadchip microarray platform. The sequencing libraries were generated with the NEBNext® UltraTM RNA Library Prep Kit for Illumina® (NEB, USA). We obtained 150 bp paired-end reads by sequencing the library preparations on an Illumina Novaseq platform. We calculated fragments per kilobase of transcript per million fragments mapped (FPKM) for the whole 58736 genes.

### Immunohistochemistry (IHC)

Immunohistochemical staining was carried out as described previously [50]. Briefly, the CCA tissue and adjacent precancerous tissue samples were stained with the primary mouse anti-human OSM antibody (1:20 dilution; Cat. No. NBP1-47904; Novus Biologicals, USA). The staining intensity of the samples was scored as follows: negative, 0; weak, 1; moderate, 2; strong, 3. The proportion of positive cells in each sample were scored as follows: negative, 0; < 5% positive tumor cells, 1; 5-25% positive tumor cells, 2; 25-50% positive tumor cells, 3; > 50% positive tumor cells, 4. The IHC scoring was independently evaluated by two experienced pathologists.

### Western blotting

Total tissue protein lysates were prepared from the snap-frozen CCA patient samples using the RIPA buffer (PC101, EpiZyme, shanghai, China). The protein concentrations were determined using the BCA protein assay. Then, equal amounts of protein samples were separated on SDS-PAGE and transferred onto PVDF membranes. Then, the membranes were blocked with 5% skimmed milk for 1 h at room temperature. Then, the membranes were incubated with primary mouse anti-human OSM antibody (NBP1-47904, Novus Biologicals, USA, 1:1000) and anti-GAPDH antibody (Cell Signaling Technology, Danvers, MA, USA, 1:1000) overnight at 4°C. Then, the membranes were incubated with the horseradish peroxidase (HRP)- conjugated secondary antibodies (Cell Signaling Technology, Danvers, MA, USA, 1:5000) at room temperature. Then, the blots were developed with the ECL system (Thermo Fisher Scientific, MA, USA).

### RNA-seq data processing

We used the R software package (v.3.5.3 and v.3.6.1; https://www.r-project.org) to analyze the RNA transcriptome and somatic nucleotide variation data. We identified differentially expressing genes (DEGs) between the precancerous and CCA tissues as well as the wild-type *TP53* vs. mutant *TP53* groups of CCA patients using the edgeR package (3.18.1). The P values for the differences in gene expression between precancerous and CCA tissues were adjusted using the Benjamini and Hochberg method, and genes with |logFC| ≥1 and adjusted p-value <0.05 were filtered and designated as differentially expressed. Furthermore, the heat maps of the DEGs were visualized using the pheatmap R package, and the volcano plots were generated using the tools in the online sangerbox (http://sangerbox.com/Tool). The overlapping DEGs were visualized with the Venn diagram generated using the Bioinformatics and Evolutionary Genomics website (http://bioinformatics.psb.ugent.be/webtools/Venn/).

### Estimation of tumor infiltrating immune cells

We used the TIMER (http://timer.cistrome.org/) database [51] and CIBERSORT algorithm [24, 52] to estimate the proportions of 22 different types of tumor-infiltrating immune cells in 5 pairs of CCA samples from our CCA patient dataset and the GSE32225 dataset. The proportions of 22 immune cell subtypes in CCA tissues, correlations, and the principal component analysis (PCA) were visualized using the Vioplot, corrplot and ggplot2 R packages, respectively.

### Analysis of somatic mutations

The single nucleotide variants (SNVs) in genes from the CCA tissues in the TCGA dataset were analyzed with the VarScan R packages [49]. The SNVs for the CCA tissues from the TCGA and ICGC datasets were visualized using maftools [53] and GenVisR [54] packages, respectively. The overlapping genes with somatic mutations were analyzed using the Kaplan-Meier survival curves and the log-rank test at the GEPIA website (http://gepia.cancer-pku.cn/).

### Functional enrichment analysis

The altered pathways and biological functions in the CCA tissues were determined by performing functional enrichment analysis of the overlapping DEGs in the Sun Yat-Sen University CCA patient cohort, and the GSE32225 and TCGA datasets using the Metascape (https://metascape.org/) database [55]. The functional enrichment analysis (GO and KEGG pathways) of the overlapped DEGs between the wild-type TP53 and mutant TP53 containing CCA patient tissues were performed using the Broad Institute GSEA software 4.0. P <0.05 was used as the threshold to identify enriched GO terms in the cellular component (CC), molecular function (MF), and biological process (BP) categories. The results of the KEGG pathway and GO analyses were visualized using the clusterProfiler [56] and ggplot2 R packages, respectively.

### Protein-protein interaction (PPI) network construction and co-expression module analyses

We used the String version 11.0 software (http://string-db.org/) to generate PPI networks of the DEGs [57]. The co-expression modules were visualized using the Cytoscape v.3.7.2 software [58]. The important modules and the top10 nodes in the PPI network were selected by calculating the node scores.

### Statistical analysis

Statistical analyses were performed with the R software (v.3.5.3 and v.3.6.1) and IBM SPSS statistical software (version.25). The Kaplan-Meier survival curves and the log-rank tests were performed using the survival R package. The correlations between the clinicopathological characteristics and the OSM expression levels were analyzed using the chi-square test, Wilcox test, univariate and multivariate Cox regression analyses. P < 0.05 was considered statistically significant.

## Supplementary Material

Supplementary Figures

Supplementary Tables
