# Supplementary material for: Oncostatin M expression and TP53 mutation status regulate tumor-infiltration of immune cells and survival outcomes in cholangiocarcinoma
Source: Aging (Albany NY). 2020 Nov 7;12(21):21518–43. doi: 10.18632/aging.103936 (PMC11623973; doi:10.18632/aging.103936)
Supplement: Supplementary Figures [file aging-12-103936-s001.pdf]

SUPPLEMENTARY FIGURES

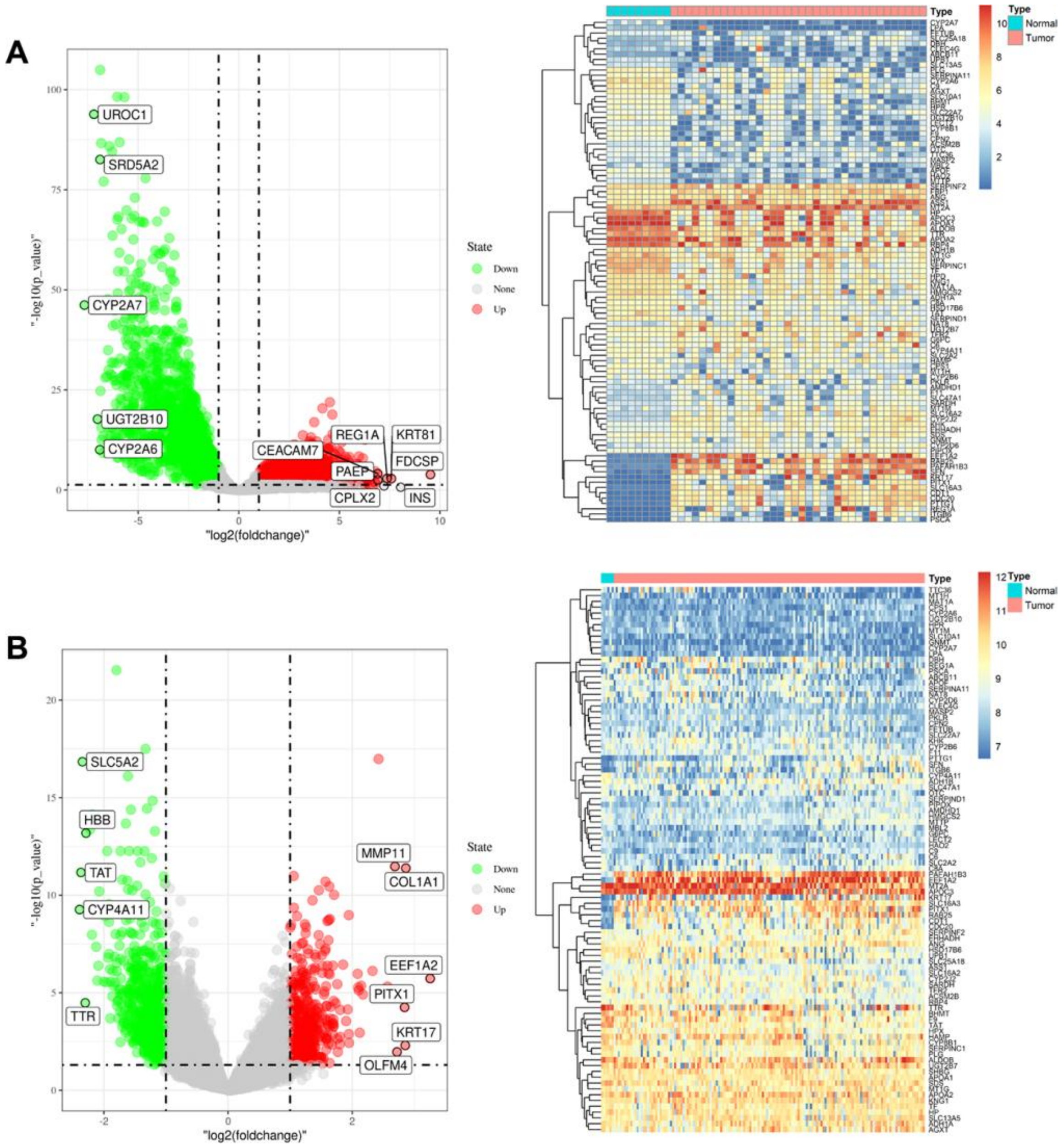

Supplementary Figure 1. (**A**) Volcano plot and heatmap of TCGA CCA and precancerous tissues. (**B**) Volcano plot and heatmap of GSE32225 CCA and precancerous tissues.

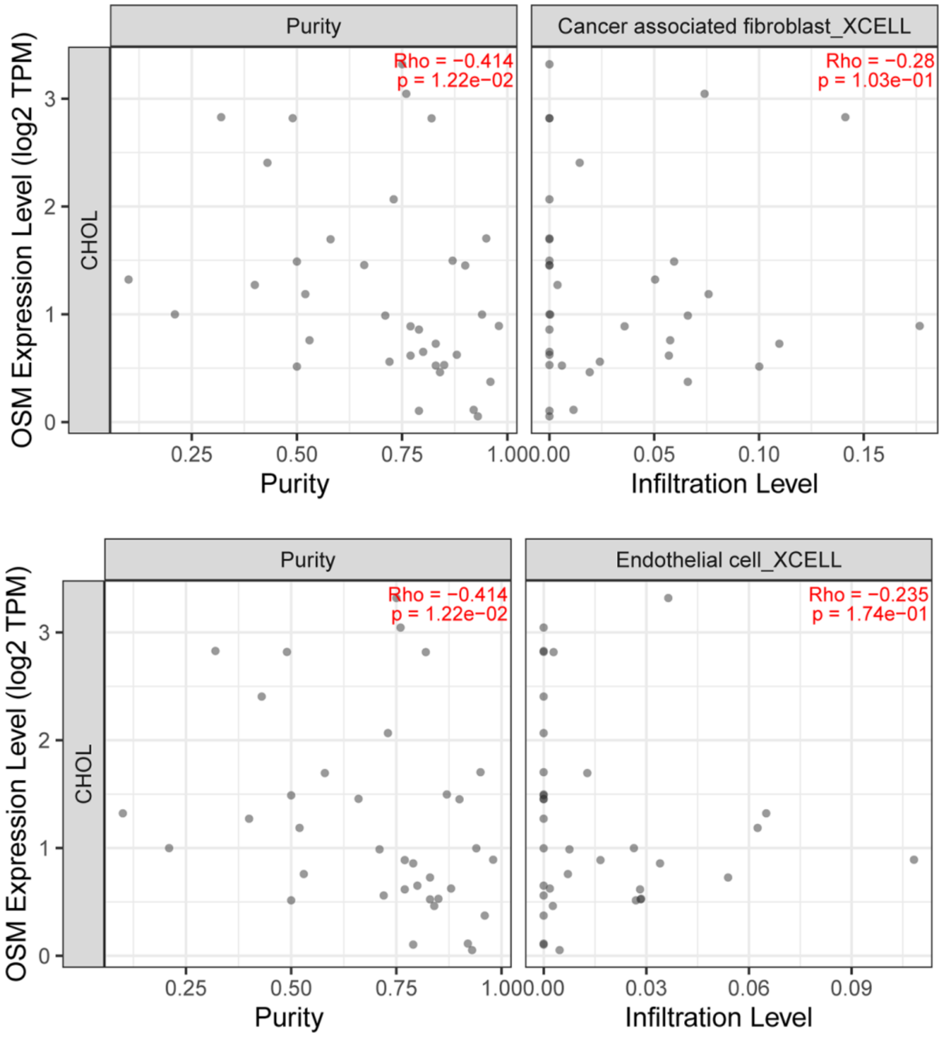

Supplementary Figure 2. Correlation between OSM expression and the proportion of cancer-associated fibroblasts and endothelial cells.
