# Supplementary material for: Oncostatin M expression and TP53 mutation status regulate tumor-infiltration of immune cells and survival outcomes in cholangiocarcinoma
Source: Aging (Albany NY). 2020 Nov 7;12(21):21518–43. doi: 10.18632/aging.103936 (PMC11623973; doi:10.18632/aging.103936)
Supplement: Supplementary Tables [file aging-12-103936-s002.pdf]

SUPPLEMENTARY TABLES

Supplementary Table 1. Correlations analysis between OSM-related immune infiltrating cells monocyte, dendritic cell, Tfh cell and four immune regulatory checkpoints CTLA4, HAVCR2, LAG3, PDL1 by TIMER.

Cell type	Gene marker	CTLA4		HAVCR2		LAG3		PDL1	
		Cor	P	Cor	P	Cor	P	Cor	P
Monocyte	CD86	0.650	*	0.794	*	0.466	*	0.456	*
	CD115(CSF1R)	0.184	0.291	0.606	*	0.033	0.853	0.315	0.065
Dendritic cell	HLA-DPB1	0.658	*	0.549	*	0.484	*	0.368	*
	HLA-DOB	0.610	*	0.320	0.0613	0.490	*	0.413	*
	HLA-DRA	0.647	*	0.581	*	0.534	*	0.389	*
	HLA-DPA1	0.663	*	0.641	*	0.556	*	0.393	*
	BDCA-1(CD1C)	0.543	*	0.427	*	0.180	0.30	0.224	0.196
	BDCA-4(NRP1)	0.188	0.279	0.402-	*	-0.028	0.873	0.413	*
	CD11C(ITGAX)	0.383	*	0.770-	*	0.193	0.266	0.275	0.110
Tfh cell	BCL6	0.358	*	0.195	0.262	0.089	0.611	0.585	*
	IL21	0.285	0.097	0.139	0.427	0.173	0.321	0.230	0.183

Supplementary Table 2. Summary of clinic parameters of CCA patients collected for RNA-seq and western-blotting.

Sample number	Age years	Gender	Grade	CA125 U/mL	CA199 U/mL	CEA ug/L	AFP ug/L	Size cm
1	74	male	moderate	36.40	47.35	5.40	3.90	2
2	59	female	moderate	8	20.29	0.58	1.23	2
3	62	male	moderate-low	479	>12000	422.43	1.56	8
4	59	male	moderate	16.4	241.08	7.82	2.21	4.5
5	61	male	moderate	58.4	2281.71	12.41	2.85	6
6	36	male	moderate	8.70	10.67	3.17	12.04	5
7	51	female	moderate-low	37.20	<2.00	2.21	2.14	8.5
8	53	female	moderate	40.90	1080.58	2.53	1.84	2.5
9	65	male	moderate	26.10	>12000	9.80	5.08	5.5
10	49	male	moderate-low	2490.60	>12000	34.92	3.25	5.5
11	52	female	moderate-low	26.60	3262.91	2.17	2.83	4.5
12	38	male	moderate	-	1174.2	-	-	5
13	58	female	moderate	5	6.53	1.09	3.21	6
14	48	male	moderate	10.60	800.71	528.70	2.66	3
15	82	male	moderate-high	62.80	118.14	4.15	18.01	5
16	53	male	moderate	2.9	<2.00	3.20	4.03	4
17	65	male	moderate	11.50	<2.00	1.79	1.51	5
